# Editorial Note

**Published:** 1918-03

**Authors:** 


### The Medical Bulletin

SUPPLEMENT PARIS MARCH 1918

### EDITORIAL NOTE

In response to a request made by the Research Committee of the American Red Cross Society, the Medical Bulletin is publishing in full the papers read at the February Meeting of the Research Society on Suture of War Wounds. This departure from the usual practice of abstracting the reports made before the Society, has necessitated the issue of the present advance supplement to the March number of the Bulletin. It contains the papers on Suture presented by the French speakers. Those read by the British surgeons will appear in the March number of the Bulletin. Because of unavoidable delays it has been impossible to print the two sets of papers together. To economize space in the March number an abstract of an article on Osteomyelitis is also included in this supplement.

